# Nitrous Oxide Production and Hydroxylamine Accumulation in a Partial Nitritation Sequencing Batch Reactor: Comparison of Different Operational Strategies

**DOI:** 10.1021/acsestwater.5c00856

**Published:** 2025-12-20

**Authors:** Lluc Olmo, Julián Carrera, Julio Pérez

**Affiliations:** GENOCOV Research Group, Department of Chemical, Biological and Environmental Engineering, School of Engineering, Universitat Autònoma de Barcelona, Ed. Q-Campus UAB, 08193 Bellaterra, Barcelona, Spain

**Keywords:** ammonia-oxidizing bacteria, reject water, carbon footprint, microaerobic

## Abstract

Sequencing batch reactors (SBRs) performing partial nitritation (PN) for treating high-strength ammonium wastewater are known to exhibit elevated levels of nitrous oxide (N_2_O) emissions. This study investigated N_2_O production and hydroxylamine accumulation in a PN-SBR operated using three distinct strategies. The N_2_O emission factor (EF) and net production rate (N2OR) were determined under stable conditions for (i) single feeding with continuous aeration and one microaerobic stage before settling (strategy I), yielding EF = 4.4% and N2OR = 14 mg N g^–1^ VSS d^–1^; (ii) single feeding with multiple microaerobic stages distributed throughout the cycle (strategy II), yielding EF = 13.5% and N2OR = 85 mg N g^–1^ VSS d^–1^; and (iii) step feeding with one single microaerobic stage before settling (strategy III), yielding EF = 10% and N2OR = 45 mg N g^–1^ VSS d^–1^. The distribution of microaerobic stages throughout the cycle (strategy II) promoted the highest hydroxylamine accumulation (0.18 mg N L^–1^) during the aerated stage, whereas strategy I showed the lowest accumulation (0.01 mg N L^–1^). A strong positive correlation (*R* ^2^ ≥ 0.9) was observed among the specific ammonium oxidation rate (AOR), specific N2OR, and bulk liquid hydroxylamine concentration during the aerated stages.

## Graphical abstract

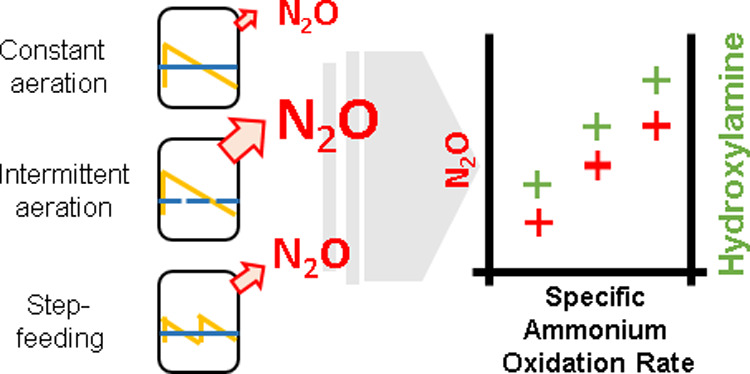

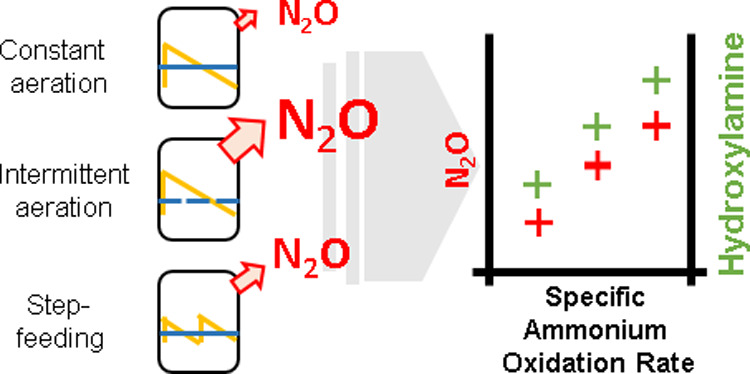

## 1 Introduction

Nitrous oxide (N_2_O) is emitted during wastewater treatment processes and contributes significantly to the greenhouse gas footprint.^1,2^ N_2_O production is linked to conventional biological nitrogen removal (BNR) processes, where the possible N_2_O sources are the activity of ammonia-oxidizing bacteria (AOB) and heterotrophic denitrifying bacteria.^3^ N_2_O production can be elevated during the autotrophic BNR process, particularly in the case of partial nitritation (PN) plus Anammox applied to the side-stream treatment of reject water from the dewatering of digested sludge. PN is considered the major contributor to N_2_O formation in these processes,^4^ primarily due to the low carbon-to-nitrogen (C/N) ratio in reject water. The low C/N ratio and aerobic conditions of the process limit the involvement of heterotrophic denitrifying bacteria in N_2_O production.^5–7^ Moreover, Anammox bacteria lack the enzymatic machinery needed for N_2_O production.^8^ This suggests that within an autotrophic BNR process, the primary source of N_2_O is the AOB population,^9^ known for its diverse nitrogen oxidation and reduction pathways encoded in their genomes.^10^ AOB metabolism enables adaptability to diverse environmental conditions and can produce N_2_O through two main pathways: hydroxylamine oxidation and nitrifier denitrification.^11–13^ Both pathways dissipate electrons for growth and maintenance, depending on redox conditions, causing the need for oxidation or reduction reactions.^14^ Differential N_2_O production observed across various AOB genera underscores the need for detailed enzymatic characterization in mixed cultures.^15,16^ The current lack of comprehensive data on intermediates such as hydroxylamine and nitric oxide represents a major limitation, constraining a complete understanding of the underlying mechanism.^17–19^ The nitritation intermediates should be considered when characterizing PN processes in order to feed existing dynamic models for understanding and tracing proper mitigation strategies under the complexity of AOB cultures.^20–22^ However, accumulation of intermediates, like hydroxylamine, during the PN process can take place on a smaller scale than other N-species (ammonium, nitrite, and nitrate), and its influence on N_2_O accumulation might have been underestimated in previous studies.^23^

Autotrophic BNR, understood as PN plus Anammox, can be implemented with different configurations, including one- or two-stage processes.^24^ In two-stage configurations, sequencing batch reactors (SBRs) have been proposed as an interesting technological alternative for the PN process.^25–27^ Indeed, SBRs facilitate the implementation of operational strategies that improve process stability and effectively repress nitrite-oxidizing bacteria (NOB).^28,29^ Among the most frequently applied operational strategies are step feeding and intermittent aeration.^30–32^ However, some of these operational strategies cause the formation and emission of N_2_O. On the one hand, Su et al.^33^ reported N_2_O emissions during the application of the step-feeding strategy in a PN-SBR, finding that transient ammonium overloading in the bulk liquid increased the N_2_O emissions linked to transient hydroxylamine accumulation after each feeding pulse. They suggested that accumulated hydroxylamine was later oxidized to N_2_O, as previously proposed by Chandran et al.^34^ Generally, the step-feeding strategy has been considered effective in reducing N_2_O emissions over long-term operation.^21,35^ On the other hand, the transition from microaerobic conditions (below 0.2 mg O_2_ L^–1^) to fully aerobic conditions once the aeration restarts is intrinsic to PN-SBR systems, and this situation can be maximized during the intermittent aeration strategy implementation.^36^ Intermittent aeration was studied by Domingo-Félez et al.,^37^ who concluded that increasing the aeration frequency mitigated N_2_O production. However, N_2_O emissions from intermittent aeration remain debatable^38^ since multiple processes occur simultaneously during the application of this strategy (e.g., biomass settling, anoxic conditions, transition from microaerobic to aerobic, etc.). Rodriguez-Caballero et al.^39^ found that the settling stage accounted for 60–80% of the N_2_O emissions in a PN-SBR since N_2_O accumulated in the bulk liquid during the settling stage and was stripped out once the following cycle began. Furthermore, a rapid increase in the specific ammonium oxidation rates during the transition from anoxic (or microaerobic) to aerobic conditions has been linked to transient hydroxylamine accumulation and subsequent N_2_O formation via hydroxylamine oxidation pathways.^17,34^ In fact, Yu et al.^40^ demonstrated that a *Nitrosomonas* culture showed cellular adaptation (specifically, an increased hydroxylamine turnover capability) when exposed to anoxic–oxic cycling. In the cited studies, the term ‘anoxic’ indicates that oxygen removal was achieved through N_2_ bubbling, with nitrite serving as the sole electron acceptor.

Moreover, the production of N_2_O in the PN-SBR cycle does not occur exclusively under transient conditions caused by the implementation of step feeding or intermittent aeration strategies. Indeed, N_2_O can be produced and emitted during the stable aerated periods due to the achievement of high ammonium oxidation rates that cause hydroxylamine accumulation (among others) in AOB-enriched cultures.^13,41^

The dynamics of hydroxylamine depletion in PN-SBRs operating with either step feeding or intermittent aeration strategies have been studied separately.^40–42^ However, no studies have compared the combined effects of the most common operational strategies applied in SBRs to achieve partial nitrification-intermittent aeration and step feeding in the long-term N_2_O production and emissions. In this study, the separate determination of N_2_O production in each stage of the SBR cycle and the establishment of correlations between the bulk liquid hydroxylamine concentration, specific ammonium oxidation rate, and N_2_O production rate were also addressed. Hence, the objective of this work was to identify which operational strategy results in the highest N_2_O production and which stage of the SBR cycle contributes most to N_2_O emissions during extended operation under stable conditions in a PN-SBR treating high-strength ammonium wastewater.

## 2 Materials and Methods

### 2.1 Sequencing Batch Reactor Configuration and Operation

A stainless-steel reactor with a working volume of 20 L was inoculated with sludge from an urban WWTP (Catalonia, Spain). The reactor was operated in the SBR mode, treating synthetic N-concentrated wastewater (ca. 300 mg N-NH_4_^+^ L^–1^) with a volume exchange ratio of 50%. The detailed composition of the synthetic wastewater can be found in the Supporting Information. The pH, temperature, and dissolved oxygen (DO) were measured using online sensors. The pH was controlled and maintained at 7.8 ± 0.1 throughout the experimental campaign by dosing a 2 M KHCO_3_ solution. The temperature was maintained at 20 ± 1 °C by manually adjusting the heat exchange coil temperature when needed. Compressed air was supplied through an air diffuser placed at the bottom of the reactor at a flow rate of 105 L h^–1^ during the aeration stage of the SBR cycle. Samples were periodically withdrawn from the reactor for further analysis of dissolved nitrogen compound concentrations, biomass concentrations, settling velocity tests, and 16S rRNA analysis. The cycle length was adapted during the start-up period to maintain an ammonium concentration of 90–120 mg N-NH_4_^+^ L^–1^ in the effluent. The cycle stage distributions for each operational period are explained below. This ammonium concentration was chosen considering that the PN effluents should be the influent of an Anammox reactor, and therefore, approximately 60% of the ammonium entering the PN-SBR (300 mg of N-NH_4_^+^ L^–1^) would be oxidized to nitrite, while the rest would remain as ammonium.

The stability of the performance was evaluated using the coefficient of variation (CV)^43^ determined for the operational parameters reported in this study (Table 1). To establish the stable period for each imposed operational strategy, we chose the most suitable performance indicators. A threshold of CV ≤ 10% sustained over at least 10 consecutive hydraulic retention times (HRTs) when performing PN (nitrate effluent concentration below 10 mg N L^–1^) was applied as the primary criterion for identifying stable period conditions. The resulting stable periods for each strategy studied are start-up (from days 118 to 127), strategy I (from days 145 to 174), II (from days 196 to 221), and III (from days 309 to 338). The biomass concentration from total suspended solids (TSS) and volatile suspended solids (VSS) was determined according to standard methods.^44^ Complementarily, the Sludge Volume Index (SVI) of 1 L of biomass was determined at 30 min in a graduated cylinder.

**Table 1:** Mean, Standard Deviation, and Coefficient of Variation (CV) of Variables during Stable Periods for Each Implemented Strategy

parameter	mean value ± standard deviation				^a^coefficient of variation (%)				operational days			
operational strategy	start-up (*n* = 4)	I (*n* = 7)	II (*n* = 6)	III (*n* = 6)	start-up	I	II	III	start-up	I	II	III
NLR (g N L^–1^ d^–1^)	0.59 ± 0.05	0.58 ± 0.03	0.55 ± 0.03	0.57 ± 0.01	8.5	5.2	5.5	1.8	118 to 127	145 to 174	196 to 221	309 to 338
AOR (g N L^–1^ d^–1^)	0.36 ± 0.02	0.33 ± 0.02	0.40 ± 0.04	0.36 ± 0.02	5.6	6.1	10	5.7				
sAOR (g N g^–1^ VSS d^–1^)	0.26 ± 0.01	0.27 ± 0.02	0.67 ± 0.06	0.58 ± 0.01	3.8	7.4	8.9	1.7				
DO (mg O_2_ L^–1^)	1.4 ± 0.2	1.10 ± 0.08	0.68 ± 0.05	0.77 ± 0.06	9.3	7.3	7.1	7.5				
N-NH_4oxidized_^+^ (%)	66 ± 4	62 ± 6	68 ± 6	63 ± 1	6.1	9.7	8.8	1.6				

a Coefficient of variation (CV) = standard deviation/mean *100.

Ammonium, nitrite, and nitrate concentrations in the influent and effluent were regularly measured off-line with both Hach Lange test kits (Hach Lange, Germany) and ionic chromatography using ICS-2000 (DIONEX Corporation) in previously filtered (0.22 μm pores) samples. Hydroxylamine was measured spectrophotometrically after pretreatment with sulfamic acid.^23,45^ The nitrous oxide in the liquid was measured using a Clark-type sensor (Unisense, Denmark). Nitrous oxide concentration in the gas phase was estimated based on the volumetric mass transfer coefficient (*k*_*L*_ *a*_*N*2_ *_O_*), as detailed in Supporting Information.

### 2.2 Cycle Stage Configuration and Operational Strategies

An extended characterization of N_2_O production during the PN-SBR operation was carried out for 338 days. The total length of the SBR cycles was constant (6.5 h) throughout the study, corresponding to a hydraulic retention time (HRT) of 0.54 days. However, different cycle stages were established to assess the effect of several operational strategies on N_2_O production (Figure 1). First, a cycle composed of a single feeding, a sole aerated stage (5.5 h), and a subsequent settling stage (1 h) was implemented during the start-up. The aim of the start-up period was to achieve a stable PN in the SBR. After reaching a stable operational period (from days 118 to 127), the effect of reducing the aerobic-to-total cycle time ratio was investigated during subsequent operational periods. For strategy I (from days 128 to 180), the modification of the cycle consisted of reducing 1 h of the aerated stage and adding a microaerobic stage of 1 h before settling. This proportion of the aerobic-to-total cycle time ratio was kept constant throughout strategies I, II, and III. This microaerobic stage was established by turning off aeration and maintaining mechanical stirring (600 rpm), and was characterized by the absence of aeration, resulting in oxygen transfer solely through the static water surface. The dissolved oxygen concentrations during this stage were consistently maintained below 0.2 mg O_2_ L^–1^. Strategy II (from day 181 to 270) consisted of the distribution of the microaerobic stage of strategy I in three separate periods throughout the cycle, but maintaining the same total microaerobic time of 1 h. Consequently, strategy II was characterized by the application of an intermittent aeration strategy. Finally, strategy III mimicked the stages of strategy I but divided the single feeding of the previous strategies into two pulses. Therefore, strategy III (from days 271 to 338) was characterized by the application of a step-feeding strategy.

**Figure 1:**
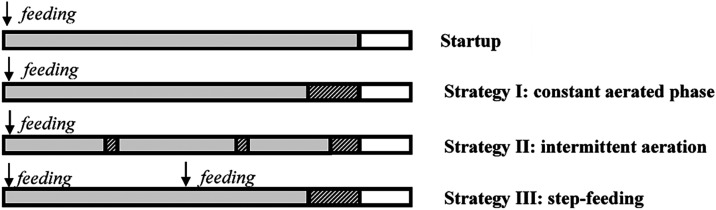
Cycle stage configuration throughout the study (start-up, strategy I, strategy II, strategy III). The gray areas represent the aerated stages of the cycle. The microaerobic stages were patterned when aeration was stopped, and the stirrer velocity was kept at 600 rpm. White represents the final settling and decanting stages.

### 2.3 Rates and Emission Factor Calculations

Specific nitrous oxide production rates (N2OR in mg N-N_2_O g^–1^ VSS d^–1^) as well as emission factors (EFs, in % N-N_2_O emitted per N-NH_4_^+^ oxidized) were used to quantify N_2_O production at stable reactor conditions achieved for each operational strategy.

The total N2OR was calculated as the sum of three different rates: N2OR_aer_, N2OR_peak_, and N2OR_effluent_. The N_2_O produced during the aerated stages of the cycle was quantified as N2OR_aer_, excluding the N_2_O measured in the form of a peak during the first few minutes of the aerated stages (N2OR_peak_). In start-up, strategies I and III, N2OR_peak_ is constituted by the N_2_O peak after the settling stage, but in strategy II, it is the sum of the three N_2_O peaks generated upon aeration after each microaerobic and settling stage imposed during the cycle. N2OR_effluent_ includes N_2_O present in the effluent of the SBR at the end of the cycle and represents a part of the N_2_O produced during the settling stage. Finally, the EF was calculated as the percentage of ammonium oxidized to N_2_O and emitted during the aerated stages (see Figure 2 and calculations in Supporting Information).

**Figure 2:**
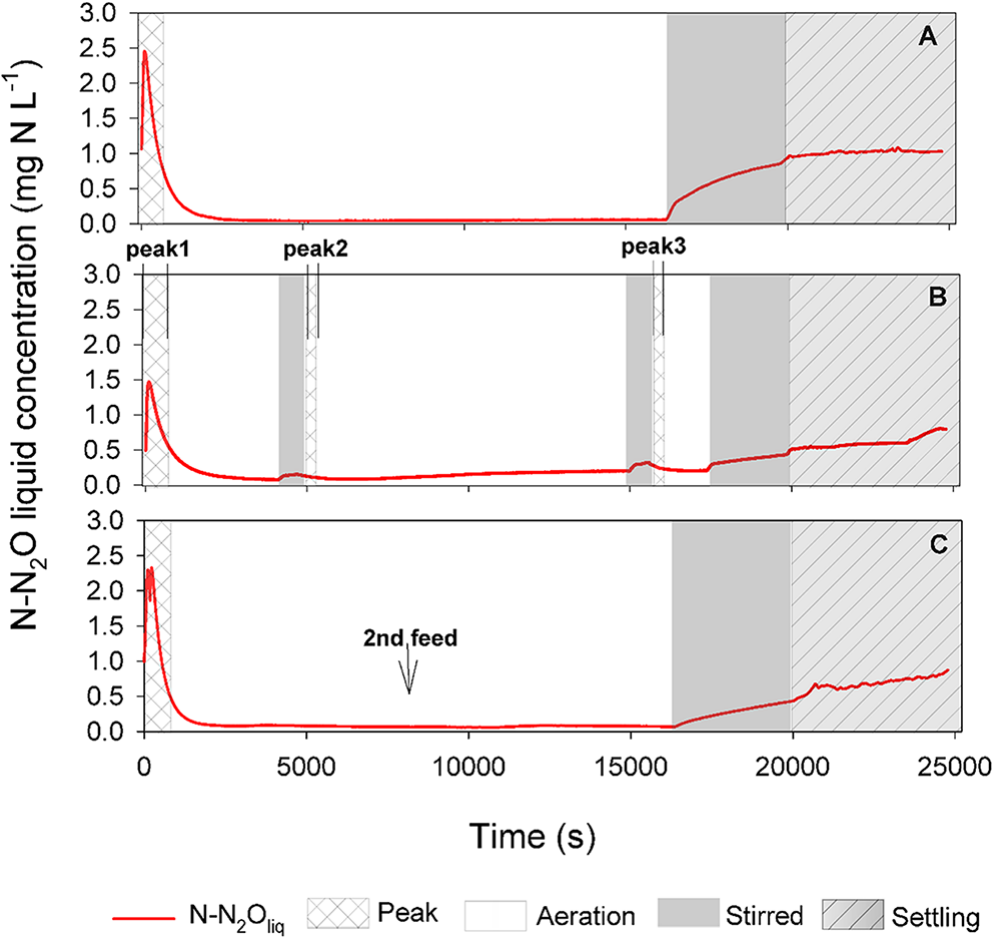
Nitrous oxide concentration in the liquid phase along the cycle across diverse operational strategies. (A) Strategy I: constant aeration and single feeding. (B) Strategy II: two aeration stops were introduced during the aerated stage. Two extra peaks contributed to the N2OR_peak_ during this strategy. (C) Strategy III: the aeration stage was continuous, and the feed was divided into two pulses.

Other parameters, such as the ammonium oxidation rate (AOR), specific AOR, and nitrogen loading rate (NLR), were also used to assess the performance of the PN-SBR. The calculations of these parameters are explained in Supporting Information.

### 2.4 Microbiological Characterization

The microbiological community composition was identified using next-generation sequencing analysis. Five biomass sampling events were carried out: (i) end of the stable period during start-up (day 127), (ii) end of the stable period for strategy I (day 174), (iii) end of the stable period for strategy II (day 218), (iv) end of strategy II conditions (day 271), and (v) end of the stable period for strategy III (day 338). The details of the protocol for DNA extraction and subsequent analysis can be found in Supporting Information.

## 3 Results and Discussion

### 3.1 Partial Nitritation Performance in the PN-SBR

The first objective of this study was to develop a stable PN system with flocculent biomass to study N_2_O production under different operational strategies. Throughout the start-up period, the specific AOR increased from low initial values (0.03 g N L^–1^ d^–1^ on day 1) after inoculation up to 0.17 g N L^–1^ d^–1^ on day 63, indicating an increase in nitrification activity. The specific AOR remained stable until day 128, when the conditions were switched to strategy I. At that point, the system achieved stable PN with approximately 60% of the influent ammonium concentration converted to nitrite and low nitrate effluent concentrations (4 ± 1 mg N-NO_3_^–^ L^–1^, Figure 3A), and this conversion was maintained throughout the reactor operation. The stable periods for each strategy studied are highlighted in Figure 3, and the mean values are reported in Table 1. The sludge retention time (SRT) was kept at 49 ± 5 d. The biomass concentration remained stable at 1.1 ± 0.3 g VSS L^–1^, and SVI averaged 57 ± 15 mL g^–1^ VSS for strategies I to III. Across the different stable periods of each strategy, the DO concentration was below 0.2 mg O_2_ L^–1^ under microaerobic stages, whereas for aerated stages, it remained at 0.8 ± 0.2 mg O_2_ L^–1^ on average (Table 1). Small variations in the DO concentration at the stable aerated stages of the cycle were attributed to changes in oxygen consumption, as the aeration flow rate was constant throughout the operation. Therefore, the differences observed in both N2OR and EF were attributed to the specific conditions associated with each of the operational strategies rather than to the effect of DO concentration during aeration, since a substantial fraction of N_2_O was produced under microaerobic conditions (Figure 4 and discussion below).

**Figure 3:**
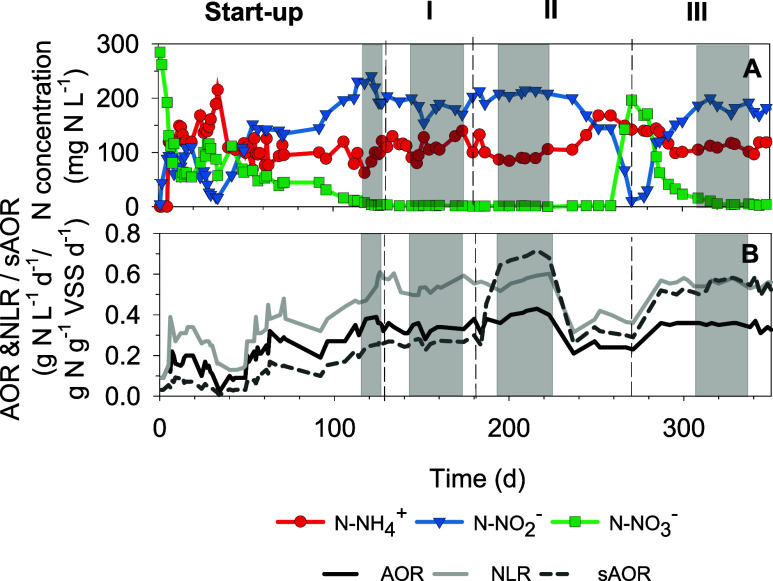
(A) N-NH_4_^+^, N-NO_2_^–^, and N-NO_3_^–^ concentrations in the effluent. The stable reactor operation periods for each operational strategy (including start-up) are marked in gray. (B) Total ammonium oxidation rate (AOR), specific AOR (sAOR), and nitrogen loading rate (NLR).

**Figure 4:**
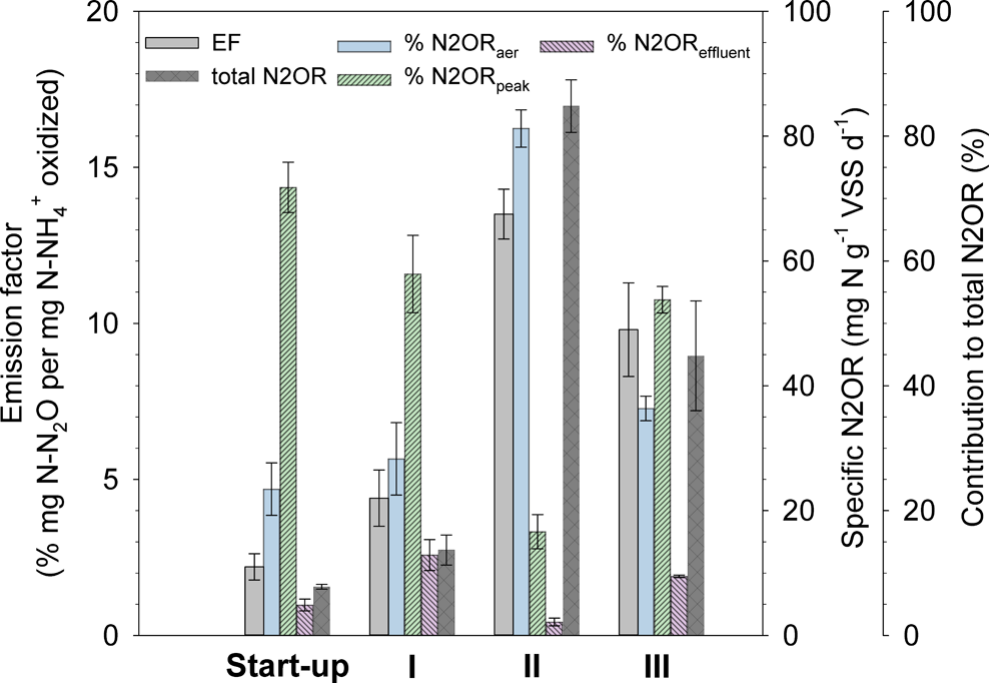
Average emission factor (EF), total specific nitrous oxide rate (N2OR), and contribution of defined N2OR_aer_, N2OR_peak_, and N2OR_effluent_ to total N2OR reported at stable reactor conditions achieved for each tested strategy, including start-up as the control.

### 3.2 Effect of Operational Strategies on N_2_O Production in the Long-Term in a PN-SBR Cycle

#### 3.2.1 EF and N2OR under Different Operational Strategies

Once stable PN performance was achieved at the end of the start-up (day 118), the EF was 2.2 ± 0.4% and the N2OR was 2.0 ± 0.5 mg N–N_2_O g^–1^ VSS d^–1^; N_2_O was mainly produced at the beginning of the cycle (see below for further explanations). These values of N_2_O production were selected as control values for comparison with N_2_O production achieved using the different tested strategies. After increasing the microaerobic time during the cycle, the EF and specific N2OR achieved at stable reactor conditions in strategy I were 4.4 ± 0.3% and 14 ± 3 mg N–N_2_O g^–1^ VSS d^–1^, respectively (see gray area for the stable reactor operation period in Figure 3 and EF and N2OR values in Figure 4). Strategy II involved dividing the total microaerobic period of strategy I into two 10-min interspersed microaerobic stages and a 40-min microaerobic stage before settling (see the scheme in Figure 1, maintaining the same total microaerobic time as applied in strategy I). This change increased the EF and specific N2OR up to 13.5 ± 0.7% and 85 ± 5 mg N–N_2_O g^–1^ VSS d^–1^, respectively (check the period in Figure 3 and EF and N2OR values in Figure 4). Moreover, these high average values were stably maintained during long-term operation (more than 25 days). However, at that point in strategy II, a significant decrease of the AOR and an increase of the nitrate concentration in the PN-SBR were detected (Figure 3). This effect was attributed to the operational conditions of strategy II that initially caused a significant increase of both AOR and N_2_O production, but also a significant decrease of AOB activity in the long-term. The change in the operational conditions of strategy III led to the recovery of AOB activity. The AOR was restored as the effluent nitrate concentration progressively decreased and the nitrite concentration in the SBR recovered to the values achieved in previous strategies (Figure 3A). At the outset of strategy III, the EF and specific N2OR were only 1.3% and 8.5 mg N-N_2_O g^–1^ VSS d^–1^ (day 278), but increased up to stable average values of 10 ± 2% and 45 ± 9 mg N-N_2_O g^–1^ VSS d^–1^, respectively (gray area in Figure 3 and EF and N2OR values in Figure 4).

The EF and specific N2OR values achieved in this study are within the range of those previously reported for other comparable PN systems (Table 2). In those previous studies, where EF and specific N2OR are reported together, EF ranges between 1.0 and 13.9, while specific N2OR ranges between 4 and 86 mg N-N_2_O g^–1^ VSS d^–1^. Although these studies investigate PN reactors operated with step feeding, continuous, or intermittent aeration, establishing a direct relationship between the operational strategy and the obtained EF or specific N2OR remains challenging because each study focuses on its applied strategy. However, the results of this study clearly demonstrate how the operational strategy implemented influences the EF and the specific N2OR obtained.

**Table 2:** Summary of Previously Reported Emission Factors (EFs) and Specific Nitrous Oxide Rates (N2OR) in Comparable PN-SBR Studies Grouped by Operational Strategies^a^,^b^,^c^,^d^,^e^

			experimental conditions			
operational strategy	emission factor(%)	specific N2OR (mg N–N_2_O g^–1^ VSS d^–1^)	DO (mg O_2_ L^–1^)	SRT (d)	T (°C)	refs
continuous aeration	2.8	n.r.	4–6	9	20	^46^
continuous aeration	2.4–10.6	17–46	0.5–3	15	22–23	^47^
continuous aeration	0.4–1.2	n.r.	0.3–5	10–30	23	^48^
intermittent aeration	0.8–6.6	n.r.	1–2	26	15	^49^
intermittent aeration	2.2–4.8	n.r.	2	n.r	24	^50^
intermittent aeration	7.0–13.9	4–37	n.r	100	30	^37^
intermittent aeration + step feeding	1.0	4–12	0.5–0.8	20	33	^51^
step feeding	2.8–3.9	n.r.	1.5–2	15	22–23	^35^
step feeding	1.7–7.4	10–87	0.3–0.8	20	20–26	^33^
continuous aeration	4.4 ± 0.3	14 ± 3	0.8 ± 0.2	49 ± 5	20 ± 1	this study
intermittent aeration	13.5 ± 0.7	85 ± 5	0.8 ± 0.2	49 ± 5	20 ± 1	
step feeding	10 ± 2	45 ± 9	0.8 ± 0.2	49 ± 5	20 ± 1	

a n.r.: not reported.

b DO corresponds to the dissolved oxygen concentration during the aerobic phase.

c SRT refers to the sludge retention time.

d T is the temperature of the reactor.

e This study was performed using a one-stage partial nitritation/anammox sequencing batch reactor.

On the one hand, previous studies have identified that during intermittent aeration strategy implementation, microaerobic stages are the primary source of N_2_O in PN-SBR systems.^5,12,42^ However, the effect of intermittent aeration remains unclear.^38^ Herein, the experimental conditions imposed in strategy II of the operation triplicated the EF value from strategy I. Moreover, strategy II caused an increase in the specific AOR at the initial stages of the period (Figure 3B). However, after 25 days with a high specific AOR and N_2_O production, the sudden decrease of the AOR suggested a significant loss in AOB activity, and consequently, a drop in the N2OR. On the other hand, the effect on N_2_O during the step-feeding strategy has been explored since the overloading generated when changing the ammonium concentration in the bulk liquid has been reported to cause transient N_2_O production and hydroxylamine accumulation.^34,35^ The EF obtained during strategy III was two times higher than that measured when single feeding was implemented during strategy I. These results indicate that the step-feeding strategy increases N_2_O production over single feeding when constant aeration is applied. Moreover, the strategy III conditions enabled the AOR restoration.

These results will be examined in greater detail in the next sections, with particular attention paid to the contribution of each stage of the SBR cycle and the role of the hydroxylamine intermediate in N_2_O production at stable reactor conditions.

Overall, all tested strategies produced significantly more N_2_O than the control experiment (a 6.5 h cycle with a single feed, a continuous aerobic stage, and a settling stage). The inclusion of a 1 h microaerobic stage after the start-up period (strategy I) doubled the EF and increased the specific N2OR by 7-fold. The introduction of intermittent aeration in the cycle (strategy II) increased the EF by more than 6-fold and the specific N2OR by 40-fold. Finally, step feeding (strategy III) increased the EF by more than 4-fold and the specific N2OR by 20-fold. In particular, the intermittent aeration strategy (strategy II) led to the highest EF and specific N2OR, but the conditions imposed during this period seriously compromised AOR stability and decreased the specific N2OR of the system in the long-term. The step-feeding strategy (strategy III) facilitated AOR restoration and NOB activity suppression. In addition, it exhibited a 2-fold increase in EF compared to the single feeding pulse strategy (strategy I). This elevated EF can be attributed to the enhanced specific AOR resulting from the proliferation of AOB.

#### 3.2.2 N2OR Fractioning Distribution

In addition to quantitatively assessing the effect of each operational strategy on the overall production of N_2_O, it is of interest to investigate which stages of the SBR cycle exhibit higher levels of N_2_O production. The distribution of this production can be assessed from the calculation of the previously defined fractions of N2OR (N2OR_aer_, N2OR_peak_, and N2OR_effluent_). The distribution of each fraction changed drastically among the different operational strategies (see Figure 4). Regarding strategy II, where the microaerobic time was divided into interspersed stages during aeration, the highest total N2OR was reached (85 ± 5 mg N-N_2_O g^–1^ VSS d^–1^), and the dominant fraction was N2OR_aer_ (81 ± 3% of the total N2OR, i.e., 70 mg N-N_2_O g^–1^ VSS d^–1^). This is supported by the profiles in Figure 2, in which the highest N_2_O liquid concentration across aerated stages was measured in strategy II, and consequently, producing the highest N2OR_aer_ during this period. Conversely, strategies I and III displayed a higher contribution of N2OR_peak_ (more than 50% in Figure 4) than the N2OR_aer_ fraction. This resulted from the longer continuous nonaerated stage at the end of the cycle (the sum of the microaerobic, settling, and decanting stages), triggering higher N_2_O liquid concentration initial peak values than those measured in strategy II. This is of interest because the N2OR_peak_ in strategy II was calculated as the sum of N2OR obtained during the initial peak plus the two peaks detected in the aeration resumptions over the cycle (see Figure 2 and calculations in Supporting Information). However, results from the start-up, when no extra microaerobic time was added (apart from the settling stage), showed that even with a short microaerobic time at the end of the cycle, N2OR_peak_ also dominated N2OR fractioning.

In conclusion, the operational strategies adopted in this study had a significant impact on N_2_O production throughout the PN-SBR cycle. The intermittent aeration strategy (strategy II) enhanced N_2_O emissions (when compared to start-up (control), strategies I and III), and N2OR_aer_ was the dominant fraction of N_2_O production. In contrast, N2OR_peak_ was the dominant N2OR fraction (72, 51, and 58% in the start-up, strategies I and III, respectively) when the largest continuous nonaerated stage was imposed.

### 3.3 Nitrous Oxide and Hydroxylamine Correlation with Specific AOR at Stable Aerobic Conditions during Aerated Stages of the Cycle

According to previous studies, N_2_O production at aerated stages can be influenced by several factors: AOB activity (measured as specific AOR), DO concentration, and hydroxylamine accumulation.^41,52^ In another study, the specific AOR was positively correlated with N_2_O production in PN systems.^41^ In this study, not only was specific AOR positively correlated (*R* ^2^ = 0.9) with the specific N_2_O production rate in the aerobic phase of the PN-SBR (N2OR_aer_), but specific AOR was also positively correlated (*R* ^2^ = 0.98) with the concentration of hydroxylamine accumulated in that aerobic phase. Both mathematical correlations followed increasing exponential functions, as shown by their semi-logarithmic representation (Figure 5).

**Figure 5:**
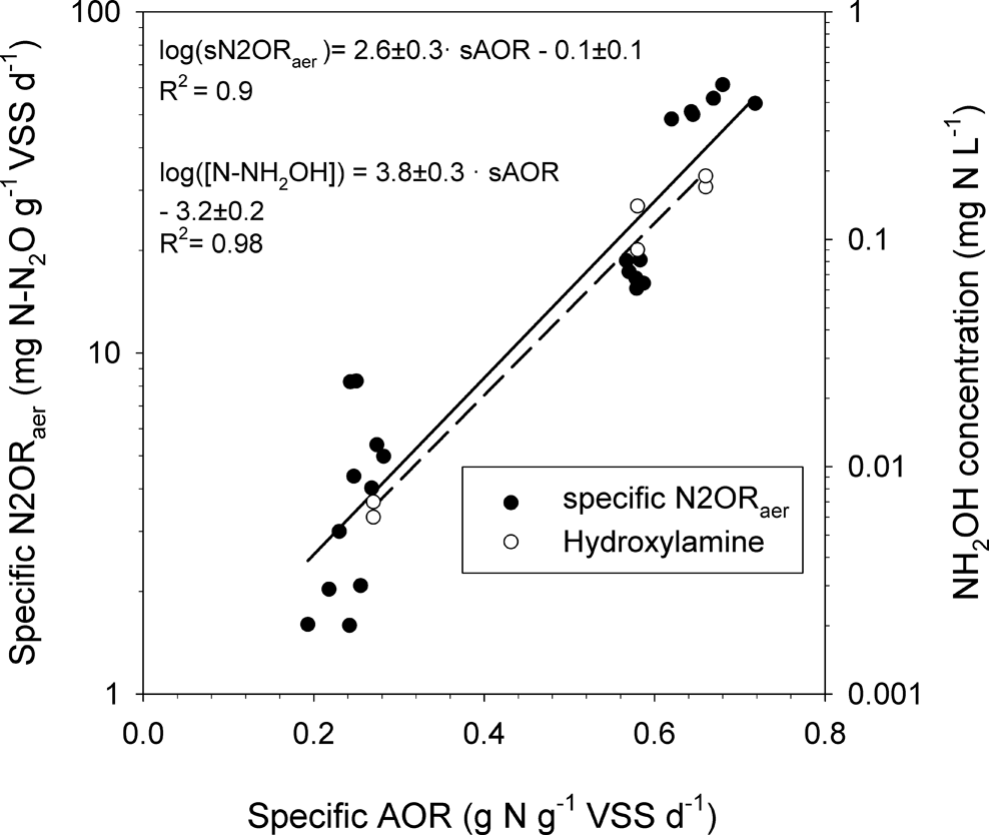
Empirical correlations showing the dependence of specific N2OR_aer_ and hydroxylamine concentrations in the liquid phase on the specific AOR during the aerobic stage of the PN-SBR.

Hydroxylamine detection in the bulk liquid of PN reactors has been associated with an imbalance in the nitrogen oxidation pathway by AOB.^34^ Hydroxylamine accumulation can potentially be attributed to high ammonium oxidation activity exceeding the hydroxylamine oxidation capacity. Thus, high specific AOR contributes to hydroxylamine accumulation depending on the culture tolerance and growth yield on hydroxylamine.^53,54^ As explained before, frequent microaerobic stages (i.e., strategy II) caused an increase in the specific AOR and specific N2OR_aer_ over an extended period of time (more than 10 HRTs). Moreover, the increased ammonium oxidation capacity to hydroxylamine caused an apparent decrease in the hydroxylamine oxidation capacity to nitrite, resulting in higher hydroxylamine accumulation during the aerated stage of this strategy. Hydroxylamine accumulation could be a potential source of N_2_O, consequently contributing to the enhancement of specific N2OR_aer_ and EF. However, prolonged application of frequent microaerobic stages led to a significant decrease in AOB activity in the long term. On the other hand, the step-feeding conditions (i.e., strategy III) resulted in the opposite effect because they promoted AOB activity and stabilized the specific AOR and specific N2OR_aer_ in the long term. Although the specific AOR of strategy III was relatively similar to that achieved in strategy II, the specific N2OR_aer_ and hydroxylamine concentrations of strategy III were significantly lower than those achieved with strategy II (Figure 5). Thus, the better coupling between the ammonium oxidation capacity to hydroxylamine and the hydroxylamine oxidation capacity to nitrite resulted in lower N_2_O production during the aerobic stage of strategy III than that during the same stage of strategy II.

### 3.4 Microbial Population in the PN-SBR

Microbial population dynamics during operation were investigated using 16S rRNA sequencing (Figure 6). At the end of the start-up period (day 127), there was a significant relative abundance (20%) of the *Nitrosomonas* genus, an AOB, responsible for ammonium oxidation to nitrite. Strategy I (day 174) caused an increase in the relative abundance of the *Nitrosomonas* genus up to 31% but remained constant throughout the strategy II performance. When interspersed microaerobic stages were removed, and a step feeding strategy was imposed (strategy III), the relative abundance of the *Nitrosomonas* genus increased up to 51% (day 338), suggesting that this genus was favored by cycles with a constant aerated stage. Interestingly, the increase of the relative abundance of AOB measured during strategy III, despite the constant floc particle size and biomass concentration, did not result in a higher AOR under stable reactor operating conditions (Figure 3). This indicates that SBR had developed a certain degree of ammonium oxidation overcapacity during the application of strategy III. There are described process advantages associated with building up overcapacity in this type of reactor.^55^

**Figure 6:**
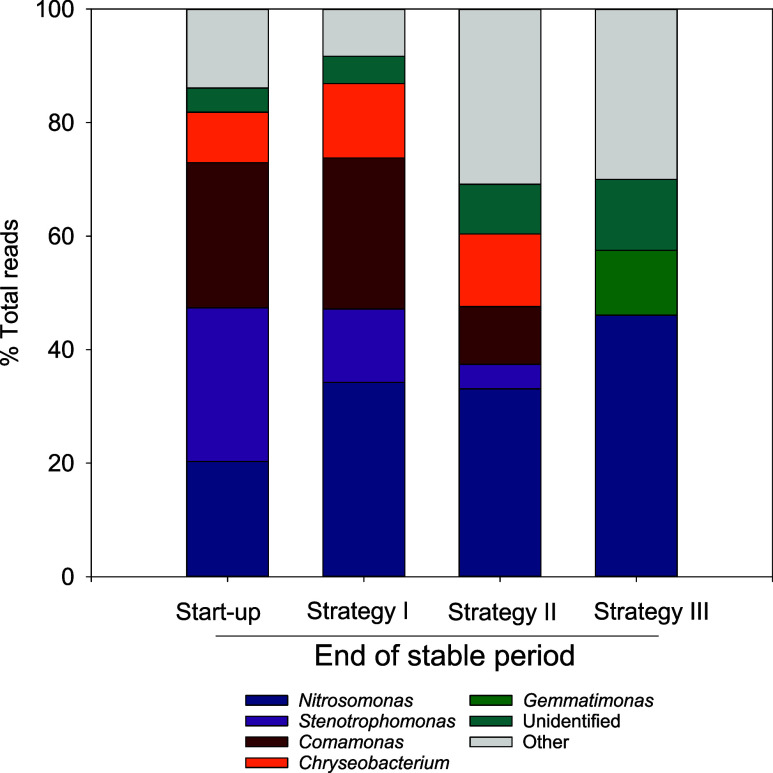
Microbial relative abundance based on 16S rRNA sequencing at the genus level. Unclassified and other fractions refer to unidentified 16S rRNA reads and those below 5% of the total reads, respectively.

No genera classified as NOB were detected, except for the *Nitrobacter* genus, which was detected only at the end of strategy II (day 271, Figure SI-2) when the effluent nitrate concentration was greater than that of nitrite. However, the relative abundance of the *Nitrobacter* genus was reduced to zero after changing the operational conditions in strategy III (day 338). Moreover, despite the lack of an organic carbon source in the synthetic wastewater, some heterotrophic genera were detected throughout the study. First, the relative abundances of *Stenotrophomonas* and *Comamonas* decreased from 27 and 22%, respectively, at the early stages of strategy I (day 174) to marginal fractions (below 5% of the total reads) during strategy III (day 338). Both genera have been reported to participate in denitrification processes by reducing nitrate and nitrite using storage polymers as electron donors.^3^ Second, the heterotrophic denitrifier *Chryseobacterium* genus,^56^ described as a protein and lipid degrader, was detected in strategies I and II (days 174, 218, and 271) with relative abundances ranging from 4 to 12% but it was not detected during strategy III (day 338). Finally, the *Gemmatimonas* genus showed up at the end of strategy II (day 271), and its relative abundance remained constant across strategy III (day 338). This genus has been studied as an N_2_O reducer^57^ and has the ability to act as an N_2_O sink even under aerobic conditions. Therefore, the heterotrophic genera found in the SBR throughout the study do not appear to have a significant effect on N_2_O production, since both their metabolic traits and the variation in their relative abundances seem to rule out this possibility.

Overall, AOB, specifically the *Nitrosomonas* genus, appeared to be the most consistent microbial population; however, the side population dynamics suggest and reveal the complexity of mixed culture system characterization in terms of N_2_O production and consumption.

### 3.5 Practical Implications of PN-SBR Systems

High-strength ammonium wastewater treatment is focused on reducing urban WWTP costs and environmental impact. Effective processes with high conversions and low-energy investments have been developed in recent years for the specific treatment of reject water. Nevertheless, these intensive conditions are known sources of N_2_O emissions (quantified as ca. 5% of the ammonium loaded), which largely contribute to the global WWTP carbon footprint.^21^ Mitigating N_2_O emissions from PN-SBR systems may require a re-evaluation of high-rate nitrogen removal processes. With regard to N_2_O abatement, the best operational conditions for PN-SBR systems appear to be single feeding and continuous aeration at low specific AORs. The application of intermittent aeration conditions causes hydroxylamine accumulation and an increase in N_2_O production along the aerated stages of the cycle. This operational strategy was also responsible for the long-term decline in AOB activity. Conversely, the step-feeding strategy causes AOB population enrichment and thus promotes high ammonium conversion. Moreover, its N_2_O production is lower than that caused by the intermittent aeration strategy. The optimization of parameters such as biomass concentration, AOB relative abundance, and proper oxygen transfer rate would be the key conditions for achieving a desired compromise between high and stable ammonium oxidation to nitrite with low N_2_O emissions in the aerated stages of the PN-SBR system. In addition, the inherent N_2_O formation during the settling and decanting stages (i.e., under microaerobic conditions) could be minimized since the longer the microaerobic stage, the higher the N_2_O concentration in both the following N_2_O peak (in the next cycle) and in the discharged effluent. Hence, operational strategies that minimize the biomass settling time (or other types of microaerobic conditions) would be a feasible approach to effectively reduce both contributions.

## 4 Conclusions

The operation of an SBR dedicated to the nitritation of wastewater with a high ammonium concentration generates significant N_2_O production. Among the most common operating strategies applied in this type of reactor, intermittent aeration is responsible for the highest N_2_O production in the long term, and most of the N_2_O is produced during the aerated stage of the cycle.

Finally, in the aerated stage of the SBR, there was a clear correlation between the accumulated hydroxylamine concentration and the specific N_2_O production and ammonium oxidation rates. A faster specific ammonium oxidation rate was associated with a larger aerobic bulk hydroxylamine concentration and higher specific N_2_O production.

## Supplementary Material



## Notes

Detailed methods for N_2_O-related calculations; hydroxylamine analysis; and additional experimental results (PDF)
