# Supplementary material for: Nitrous Oxide Production and Hydroxylamine Accumulation in a Partial Nitritation Sequencing Batch Reactor: Comparison of Different Operational Strategies
Source: ACS ES T Water. 2025 Dec 20;6(1):164–72. doi: 10.1021/acsestwater.5c00856 (PMC12798732; doi:10.1021/acsestwater.5c00856)
Supplement: Supplementary file 1 [file ew5c00856_si_001.pdf]

# Supporting information

Nitrous oxide production and hydroxylamine accumulation in a partial nitritation sequencing batch reactor: comparison of different operational strategies

Lluc Olmo, Julián Carrera*, Julio Pérez

lluc.olmo@uab.cat

julian.carrera@uab.cat

julio.perez@uab.cat

GENOCOV Research Group, Department of Chemical, Biological and Environmental Engineering, School of Engineering, Universitat Autònoma de Barcelona, Ed. Q-Campus UAB, 08193 Bellaterra, Barcelona, Spain

*Corresponding author email adress: julian.carrera@uab.cat

## Section 1 – Synthetic wastewater composition

The synthetic wastewater was adapted from [1]: 1.69 g $NH_4HCO_3$ $L^{-1}$, 0.24 g $K_2HPO_4$ $L^{-1}$, 0.24 g $KH_2PO_4$ $L^{-1}$ and 1.25 ml $L^{-1}$ of trace element solution adapted from [2] (19.11 g EDTA $L^{-1}$, 0.43 g $ZnSO_4 \cdot 7H_2O$ $L^{-1}$, 0.24 g $CoCl_2 \cdot 6H_2O$ $L^{-1}$, 1.0 g $MnCl_2 \cdot 4H_2O$ $L^{-1}$, 0.25 g $CuSO_4 \cdot 5H_2O$ $L^{-1}$, 0.22 g $(NH_4)_6Mo_7O_{24} \cdot 4H_2O$ $L^{-1}$, 0.20 g $NiCl_2 \cdot 6H_2O$ $L^{-1}$, 0.09 g $NaHSeO_3$ $L^{-1}$, 0.014 g $H_3BO_3$ $L^{-1}$and 0.054 g $Na_2WO_4 \cdot 2H_2O$ $L^{-1}$).

## Section 2 – Hydroxylamine ($NH_2OH$) analysis

The $NH_2OH$ concentration was measured following the protocol proposed by [3,4]:

1. Add 1.6 mL of the filtered sample containing $NH_2OH$ in the range of 0-2.1 mg N $L^{-1}$ to 5 mL test tube, follow with 0.2 mL of 0.1g $mL^{-1}$ sulfamic acid and mix.
2. Add 1 mL of 0.05 M phosphate buffer, 0.2 mL of 12 wt% trichloroacetic acid and 1 mL of 1% 8-quinolinol (w/v), then swirl gently.
3. Add 1.0 mL of 1.0 M sodium carbonate solution, shake vigorously.
4. Heat 1 min at 100 °C in a water bath and cool for 15 min, and then read in spectrophotometer at 705 µm.
5. Carry out simultaneously a blank by replacing the sample volume by the same volume of demineralized water.

## Section 3 - Nitrogen conversion rates

Considering aerated stages of the cycle are responsible for the reaction of N-$NH_4^+$ oxidation, aerobic ammonium oxidation rate (AOR) was defined as shown in equation SI-1:

$$AOR = \frac{(\mathrm{N-NH_4^+})_{inf} - (\mathrm{N-NH_4^+})_{eff,n}}{\mathrm{HRT}\cdot t_{aer}/t_{cycle}} - \frac{(\mathrm{N-NH_4^+})_{eff,n} - (\mathrm{N-NH_4^+})_{eff,n-1}}{t_n - t_{n-1}} \qquad \text{(Eq. SI–1)}$$

Where $(N\text{-}NH_4^+)_{inf}$ and $(N\text{-}NH_4^+)_{eff}$ are $N\text{-}NH_4^+$ concentrations on operation day *n*. The $t_{aer}$ and $t_{cycle}$ are the aerobic stage and cycle time lengths. The $t_n$ and $t_{n-1}$ represent two consecutive operation days. Both, $(N\text{-}NH_4^+)_{eff,n}$ and $(N\text{-}NH_4^+)_{eff,n-1}$ represent $N\text{-}NH_4^+$ effluent concentrations on day $t_n$ and $t_{n-1}$ respectively.

The specific AOR (sAOR) was calculated based on the AOR and the biomass concentration in the SBR as in equation SI-2:

$$sAOR = AOR/X_R \qquad \text{(Eq. SI-2)}$$

where $X_R$ corresponds to the biomass concentration in the SBR in mg VSS $L^{-1}$.

Whereas aerobic nitrogen loading rate (NLR) was calculated based on equation SI-3 considering:

$$NLR = \frac{(\mathrm{N-NH_4^+})_{inf}}{\mathrm{HRT}\cdot t_{aer}/t_{cycle}} \qquad \text{(Eq. SI–3)}$$

## Section 4 - Calculation of the $N_2O$ gas concentration

The liquid $N_2O$ concentration in the reactor is the consequence of both, net production rate and its net mass transfer rate.

The volumetric transfer rate of $N_2O$ from the liquid to the gas phase (here notated as $TR_{N-N_2O}$, $mg\,N - N_2OL^{-1}h^{-1}$) was estimated based on the $N_2O$ mass transfer

coefficient $\left(k_L a_{N_2O}\right)$. The $k_L a_{N_2O}$ was calculated with the Eq. SI–4 according to the Higbie's penetration model as described in [5].

$$k_L a_{N_2O} = k_L a_{O_2} \times \sqrt{\frac{D_{F,N_2O}}{D_{F,O_2}}} \qquad \text{(Eq. SI-4)}$$

The $N_2O$ transfer rate $(TR_{N-N_2O})$ could be defined by using the $k_L a_{N_2O}$ (Eq. SI-5), as well as using Eq. SI-6 assuming that the off-gas flow rate is equivalent to the air flow rate supplied. As $[N-N_2O]_{liq}^{*}$ , referred to the liquid concentration (mg N $L^{-1}$) in the interphase and can be defined in Eq. SI-7:

$$TR_{N-N_2O} = k_L a_{N_2O} \times \left([N-N_2O]_{liq} - [N-N_2O]_{liq}^{*}\right) \qquad \text{(Eq. SI-5)}$$

$$TR_{N-N_2O}(mg\,N-N_2OL^{-1}h^{-1}) = \frac{[N-N_2O]_{gas}\,(mg\,N-N_2O\,L^{-1})\cdot Q_{gas}(L\,h^{-1})}{V_R\,(L)} \qquad \text{(Eq. SI-6)}$$

$$[N-N_2O]_{liq}^{*}(mg\,N-N_2OL^{-1}) = [N-N_2O]_{gas}(mg\,N-N_2O\cdot L^{-1})\cdot H_{adim} \qquad \text{(Eq. SI-7)}$$

where $[N\text{-}N_2O]_{gas}$ is the nitrous oxide concentration in the off-gas in mg N $L^{-1}$; $[N\text{-}N_2O]_{liq}$ is the nitrous oxide concentration in the bulk liquid in mg N $L^{-1}$; $D_{F,N2O}$ and $D_{F,O2}$ are the diffusion coefficients of nitrous oxide and oxygen in water, respectively, $1.88\cdot10^{-5}$ and $2.2\cdot10^{-5}$ $cm^2\,s^{-1}$ at 20 ºC, respectively [6,7]; $Q_{gas}$ is the flow rate of aeration in L $d^{-1}$; $V_R$ is the volume of the reactor in L; and $H_{adim}$ is the dimensionless Henry's constant, 0.59 (aqueous concentration/ gas concentration) [8].

Substituting the Eq. SI-7 into Eq.SI-5 allowed us to solved the two equations system (Eq.SI-5 and Eq. SI-6) in order to determine the $[N-N_2O]_{gas}$.

The mass transfer coefficient for oxygen $\left(k_L a_{O_2}\right)$ was calculated based on the oxygen uptake rate (OUR) and the DO concentration in the bulk liquid of the reactor as described by equation Eq. SI–8. The OUR was calculated based on the stoichiometric oxygen requirement for the oxidation of ammonium by AOB and the production of nitrate by NOB.

$$OUR = k_L a_{O_2} \times \left([O_2]^*_{liq} - [O_2]\right) \quad \text{(Eq. SI–8)}$$

Where $[O_2]^*_{liq}$ is the oxygen saturation concentration (8.52 mg$O_2$ $L^{-1}$) estimated following [9] based on the synthetic wastewater composition used in the experiment.

## Section 5 - $N_2O$ production rate calculation

Specific $N_2O$ production rates (N2OR) were calculated from the following equations considering the diverse $N_2O$ outputs of the system:

$N_2O$ production rate during aerated stages of the cycle was divided in N2OR aerobic ($N2OR_{aer}$) and N2OR during initial $N_2O$ peak for strategies I and III ($N2OR_{peak}$ in Equation SI-10) and summing the three peaks during strategy II (named $N2OR_{peak}$ in Equation SI-11). Through the integration of the $N_2O$ liquid to gas transfer rate ($m_{N-N2O}$, $mg\ N-N_2Oh^{-1}$) over elapsed time, the mass of N emitted could be obtained. Both, $N2OR_{aer}$ and $N2OR_{peak}$ could be calculated as shown in Equations SI-9, SI-10 and SI-11.

$$N2OR_{aer} = \frac{\int_{t_{0,a}}^{t_{f,a}} m_{N-N2O} \cdot dt}{X_R \cdot V_R \cdot t_a} \quad \text{(Eq. SI–9)}$$

$$N2OR_{peak} = \frac{\int_{t_{0,peak}}^{t_{f,peak}} m_{N-N2O} \cdot dt}{X_R \cdot V_R \cdot t_{peak}} \quad \text{(Eq. SI–10)}$$

$$N2OR_{peak} = \frac{\int_{t_{0,peak1}}^{t_{f,peak1}} m_{N-N2O} \cdot dt}{X_R \cdot V_R \cdot t_{peak1}} + \frac{\int_{t_{0,peak2}}^{t_{f,peak2}} m_{N-N2O} \cdot dt}{X_R \cdot V_R \cdot t_{peak2}} + \frac{\int_{t_{0,peak3}}^{t_{f,peak3}} m_{N-N2O} \cdot dt}{X_R \cdot V_R \cdot t_{peak3}} \quad \text{(Eq. SI–11)}$$

where $t_a$ is defined in Eq. SI-12, the $t_{peak}$ in Eq. SI-10 equals to $t_{peak1}$ in Eq. SI-11 with a value of 10 min corresponding to the initial peak whereas $t_{peak2}$ and $t_{peak3}$ correspond to second and third peak during strategy II, respectively, with a value of 5 min (Figure 2 in main body). The integrating limits for $N2OR_{aer}$ (Eq. SI-9) are defined as $t_{0,a}$ (lower) and $t_{f,a}$ (upper). For $N2OR_{peak}$ (Eq. SI-10, 11), the limits re $t_{0,peak}$ (lower) and $t_{f,peak}$ (upper).

$$t_a = t_{aerobic} - t_{peak,1,2,3} \quad \text{(Eq. SI–12)}$$

Regarding the dissolved $N_2O$ in the effluent, the N2OR was calculated as follows in Eq. SI-13.

$$N2OR_{eff} = \frac{[N-N2O]_{eff} \cdot V_{ex}}{X_R \cdot V_R \cdot t_{cycle}} \quad \text{(Eq. SI–13)}$$

The $V_{ex}$ is the exchanged volume (10 L) obtained from the volume exchange ratio (0.5) and $V_R$ (20 L). Moreover, $X_R$ corresponds to the biomass concentration in the SBR in mg VSS $L^{-1}$

The total specific nitrous oxide production rate (N2OR) was calculated as follows:

$$N2OR = N2OR_{aer} + N2OR_{peak,1,2,3} + N2OR_{eff} \quad \text{(Eq. SI–14)}$$

## Section 6 - Emission factor calculation

The nitrous oxide ($N_2O$) transferred to the gas phase (i.e., emitted) was quantified through the emission factor, that was calculated using the amount of ammonium oxidized as follows (Eq. SI-15).

$$EF = \frac{\int_{t_{0,emission}}^{t_{f,emission}} N_{N-N2O} \cdot dt}{\Delta N - NH_4^+ oxidized \cdot V_{ex}} \quad \text{(Eq. SI–15)}$$

where $t_{emission}$ is the time length devoted to aeration in each cycle, therefore the main time period causing $N_2O$ emission (i.e., $N_2O$ transfer from the liquid to gas phase due to the stripping produced by active aeration) defined as the sum of $t_{peak}$ and $t_a$.

The $\Delta N - NH_4^+ oxidized$ is calculated from the ammonium converted:

$$\Delta N - NH_4^+ oxidized = \left(\mathrm{N} - \mathrm{NH_4^+}\right)_{inf} - \left(\mathrm{N} - \mathrm{NH_4^+}\right)_{eff} \quad \text{(Eq. SI–16)}$$

## Section 7 – Operation additional results

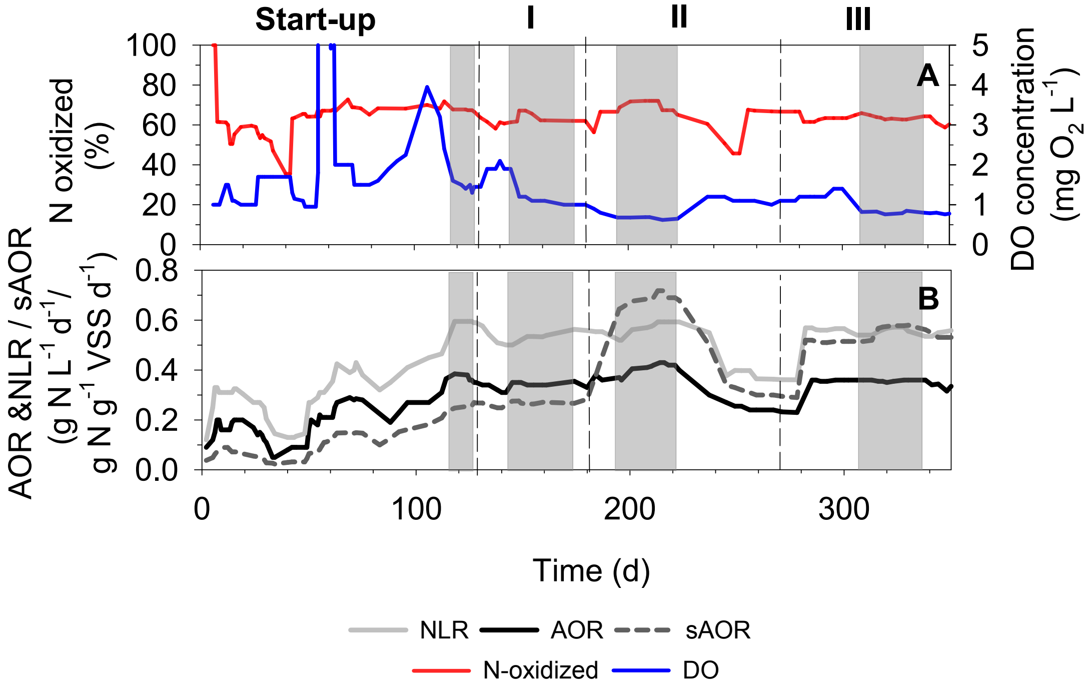


Figure SI-1. The moving average method was applied to identify potential stable periods. The mean values of each variable, averaged over three points (n=3), are plotted. A) Percentage (%) of N-NH4+ oxidized and dissolved oxygen (DO) concentration in the bulk liquid during aerated stages of the cycle. B) Nitrogen loading rate (NLR), ammonium oxidation rate (AOR) and specific AOR.

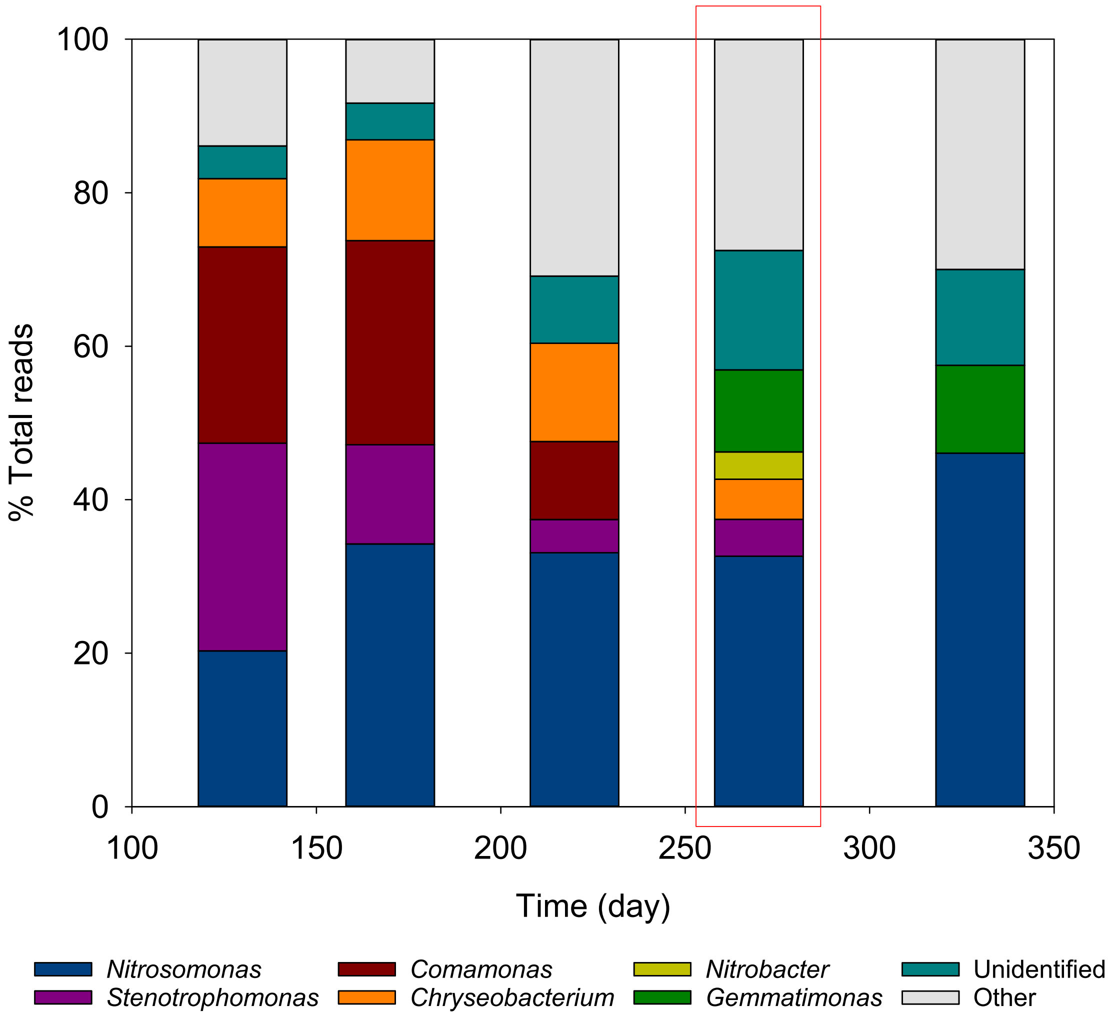


Figure SI-2. Microbial relative abundance based on 16S rRNA sequencing at genus level. Unclassified and other fractions refer to unidentified 16S rRNA reads and below 5% of the total reads, respectively. The column highlighted in red correspond to the extra sample taken at the end of strategy II. The remaining columns correspond to samples from Figure 6 in the main text.

## Section 8 - DNA extraction and next-generation sequencing analysis

DNA was extracted by using the Soil DNA Isolation Plus KitTM (Norgen Biotek Corp, Canada) following the manufacturing protocol. The quantity and quality of the extracted DNA was measured by using the NanoDrop 1000 Spectrophotometer (Thermo Fischer Scientific, USA). Absorbance 260/280 ratio of 1.9±0.1 was used as quality cut-off and a minimum of 12 ng $\mu L^{-1}$ of extracted DNA was guaranteed to perform on an Illumina MiSeq platform by the Research and Testing Laboratory (Lubbock, Texas, USA). Bacterial 16S rRNA variable regions V4 were targeted using the primer pair 515F-806R for general bacteria.

## References

[1] Kuai, L.; Verstraete, W. Ammonium Removal by the Oxygen-Limited Autotrophic Nitrification-Denitrification System, Appl Environ Microbiol 64 (1998) 4500–4506. DOI:10.1128/AEM.64.11.4500-4506.1998.

[2] Soler-jofra, A.; Wang, R.; Kleerebezem, R.; van Loosdrecht, M.C.M. Stratification of nitrifier guilds in granular sludge in relation to nitritation. Water Res 148 (2019) 479–491. DOI: 10.1016/j.watres.2018.10.064.

[3] D.S. Frear, R.C. Burrell, Spectrophotometric method for determining hydroxylamine reductase activity in higher plants, Anal Chem. 27 (1955) 1664–1665. https://doi.org/10.1021/ac60106a054.

[4] A. Soler-Jofra, B. Stevens, M. Hoekstra, C. Picioreanu, D. Sorokin, M.C.M. van Loosdrecht, J. Pérez, Importance of abiotic hydroxylamine conversion on nitrous oxide emissions during nitritation of reject water, Chem Eng J. 287 (2016) 720–726. https://doi.org/10.1016/j.cej.2015.11.073.

[5] R. Marques, A. Rodriguez-Caballero, A. Oehmen, M. Pijuan, Assessment of online monitoring strategies for measuring N2O emissions from full-scale wastewater treatment systems, Water Res 99 (2016) 171–179. https://doi.org/http://dx.doi.org/10.1016/j.watres.2016.04.052.

[6] A. Tamimi, E.B. Rinker, O.C. Sandall, Diffusion coefficients for hydrogen sulfide, carbon dioxide, and nitrous oxide in water over the temperature range 293-368 K, J Chem Eng 39 (1994) 330–332.

[7] R.T. Ferrell, D.M. Himmelblau, Diffusion coefficients of nitrogen and oxygen in water, J Chem Eng 12 (1967) 111–115.

[8] R. Sander, Compilation of Henry’s law constants (5.0.0) for water as solvent, in: Phys Atmos Chem (2023) 10901–12440. https://doi.org/doi:10.5194/acp-23-10901-2023.

[9] A. Clesceri, L.S. Greenber, A.E. Eaton, ed., Standard Methods for the Examination of Water and Wastewater, 20th ed., 1999.
